# A Node Localization Algorithm Based on Multi-Granularity Regional Division and the Lagrange Multiplier Method in Wireless Sensor Networks

**DOI:** 10.3390/s16111934

**Published:** 2016-11-18

**Authors:** Fengjun Shang, Yi Jiang, Anping Xiong, Wen Su, Li He

**Affiliations:** College of Computer Science and Technology, Chongqing University of Posts and Telecommunications, Chongqing 400065, China; jiangyi@cqupt.edu.cn (Y.J.); xiongap@cqupt.edu.cn (A.X.); huahua718422@163.com (W.S.); heli@cqupt.edu.cn (L.H.)

**Keywords:** WSN, RSSI, Voronoi diagram, vector similar degrees, Lagrange

## Abstract

With the integrated development of the Internet, wireless sensor technology, cloud computing, and mobile Internet, there has been a lot of attention given to research about and applications of the Internet of Things. A Wireless Sensor Network (WSN) is one of the important information technologies in the Internet of Things; it integrates multi-technology to detect and gather information in a network environment by mutual cooperation, using a variety of methods to process and analyze data, implement awareness, and perform tests. This paper mainly researches the localization algorithm of sensor nodes in a wireless sensor network. Firstly, a multi-granularity region partition is proposed to divide the location region. In the range-based method, the RSSI (Received Signal Strength indicator, RSSI) is used to estimate distance. The optimal RSSI value is computed by the Gaussian fitting method. Furthermore, a Voronoi diagram is characterized by the use of dividing region. Rach anchor node is regarded as the center of each region; the whole position region is divided into several regions and the sub-region of neighboring nodes is combined into triangles while the unknown node is locked in the ultimate area. Secondly, the multi-granularity regional division and Lagrange multiplier method are used to calculate the final coordinates. Because nodes are influenced by many factors in the practical application, two kinds of positioning methods are designed. When the unknown node is inside positioning unit, we use the method of vector similarity. Moreover, we use the centroid algorithm to calculate the ultimate coordinates of unknown node. When the unknown node is outside positioning unit, we establish a Lagrange equation containing the constraint condition to calculate the first coordinates. Furthermore, we use the Taylor expansion formula to correct the coordinates of the unknown node. In addition, this localization method has been validated by establishing the real environment.

## 1. Introduction

Precision agriculture is one of the most promising application domains where wireless sensor networks (WSN) may deliver a feasible or even optimal solution. Generally, wireless sensor networks consist of a large number of densely deployed small sensor nodes with sensing, computation, and wireless communication capabilities. Sensor nodes do not incorporate an infrastructure. They build up a network autonomously, without any external guidance or supervision. Precision agriculture is a crop and livestock production management system that uses a wireless sensor network to monitor equipment field positions to collect information. Precision agriculture technologies include equipment guidance and automatic steering, yield monitoring, variable rate input application, remote sensing, in-field electronic sensors, section and row control on planters, sprayers and fertilizer applicators, and spatial data management systems. Variable rate fertilizer application allows crop producers to apply different rates of fertilizer at each location across fields. The technology needed to accomplish variable rate fertilization includes an in-cab computer and software with a field zone application map, fertilizer equipment capable of changing rates during operation, and the wireless sensor network.

The Internet of Things (IoT) will connect things and people using the Internet link. It adopts intelligent recognition technology, computer communication technology, and the Internet as the core, at the level of deep development and the formation of a network of objects in a communication process to realize the exchange of information. Examples of its use are remote information management and intelligent monitoring systems [1]. From 1999, the IOT has been researched by the Chinese Academy of Sciences. Various engineering bodies in the country are using for IOT technology for deep exploration, such as the establishment of a mobile networking operations center in 2006 in Chongqing and the National Center for sensing information, established in 2009 and opened in Shanghai in 2010. IOT, as part of networking in new industries [2], is one of the key national developments. Because the sensor technology is mature and the government’s support is strong, intelligent transportation, security, and home market have been integrated into the IOT technology. Moreover, a number of cities have implemented the technology, so at present China’s IOT industry chain has been basically formed [3]. At the same time, cloud computing, big data, and the mobile Internet era have arrived. The current Internet data transfer mode changes from the traditional form to the massive data association, but the wide application of IOT promotes the development of cloud computing. Wireless sensor networks represent an enabling technology for low-power wireless measurement and control applications. The elimination of lead wires provides a significant cost savings and creates improved reliability for many long-term monitoring applications. Wireless sensor networks enable completely new capabilities for measurement and control applications.

## 2. Related Works

The main function of a wireless sensor network is collecting data. In military reconnaissance or traffic monitoring, the location information of sensor nodes is the premise for the perception, acquisition, and transmission of data. Unless they are associated with a particular position, these data will lose their significance [4]. There are many types of localization methods for WSN [5] has. According to the methods of data acquisition, processing is mainly divided into the aspects of distance, angle, time, etc. These methods acquire related positioning data by calculating and obtaining location information. According to the information processing method, no matter what kind of processing method, the aim is to convert the data to coordinate information, and finally complete the positioning function. According to the method of information processing, it is mainly divided into: range-based and range-free methods; single-hop and multi-hop algorithms; and distributed and centralized algorithms.

According to the distance of nodes in the localization process, the wireless sensor network can be divided into the positioning method based on measuring distance and the positioning method without measuring distance. The distance-based localization method [6] is divided into two steps: firstly, the information is measured by a certain method, and then the coordinates are calculated by the measured information. The information measured includes the intensity value, the transmission time (time of arrival, TOA), transmission time difference (time difference of arrival, TDOA), and azimuth angle (angle of arrival TOA). Next the computing nodes’ final position is used as the actual measurement data. The range-free positioning method [7] is firstly used to determine the range of the node, followed by a series of calculation methods to calculate the final node position.

The RSSI location method [8] uses an RF (Radio Frequency) signal, with full use of the wireless communication node. Positioning uses the wireless signal data of nodes with a loss relationship between the node transmission power and the receiving node power. It may compute the wireless signal transmission power. According to the model of the wireless signal transmission loss, the distance will be computed from the intensity data.

A TOA ranging method [9] can determine the node work starting time synchronization and calculating signal propagation time according to the relationship between the known signal propagation velocity and propagation time. Thus the distance of nodes is estimated by a time distance formula, but time synchronization cannot be explicitly guaranteed; this method has very high hardware requirements and its positioning effect is not ideal.

The TDOA location method [10] uses the signal propagation time difference between the sending and receiving nodes to estimate the distance; the prerequisite for the application of this method is that the nodes have the same properties and the propagation time of each signal must be the same, otherwise the estimated distance value will be inaccurate.

The AOA location method [11] uses the angle and azimuth of neighboring nodes to determine the relative position of the unknown node. If the hardware is not up to standard, or the power consumption is too large, this method in the application process will be interfered with by various factors, resulting in lower final positioning accuracy.

The localization algorithm based on ranging method [12,13] mainly includes: three edge location algorithm, triangulation location algorithm, maximum likelihood estimation method, etc. The existing localization algorithms based on range-free methods include the centroid localization algorithm [14], the DV-hop algorithm [15], the APIT algorithm [16], and the convex programming localization algorithm [17].

Sensor network localization relies on a large number of nodes, which via self-organization constitutes a wireless network system; the deployment of node location has a certain influence on the positioning accuracy. In order to ensure wireless sensor network communication quality and node localization efficiency as far as possible in a short period of time, the effective communication range of the reference node and the target positioning need to be optimized. The division of the regional positioning not only makes the positioning accuracy improve, but also reduces the multifarious repeat positioning calculation process.

In the division of regional positioning algorithms, the related sequence positioning algorithm is more classical and mainly includes the sequence of the localization method in WSN [18], the wireless sensor network in the sequence location algorithm [19], the N-order optimal time alignment method [20], and a Voronoi diagram of the WSN rank sequence localization method [21].

## 3. Multi-Granularity Region Partition Based on RSSI

In this paper, a multi-granularity [22] region partition method is proposed. Firstly, the location method is chosen based on the received signal strength. Due to the fluctuation of strength value, Gauss fitting is introduced to estimate the value of RSSI. Secondly, it introduces a Thiessen polygon [23] using each anchor node as the center. The whole area is divided into a plurality of sub-regions, and the sub-regions of the adjacent nodes are combined to form multiple triangles, locking the unknown node into the final area.

### 3.1. Ranging Method Based on RSSI

The distance measurement method based on the RSSI theory model includes the log normal shielding model (Shadowing Model) and the free space propagation path-loss model. The free space propagation path-loss model is not easy to use in actual environments, however, because it belongs to the ideal state transfer model. It gives the energy consumption of the signal transmission distance in an infinite vacuum, with no influence from other factors. However, in practical application, the transmission distance of the signal is not only non-linear, but also the interference from the signal is not insignificant. Considering the influence factors of various kinds of reflection, scattering, and occlusion in the practical application environment, the attenuation of the channel is similar to the log normal distribution, and the Shadowing Model is more in line with the practical application.

The Shadowing Model formula is as follows:

(1) Loss=32.44+10nlg(d)+10nlg(f),

where Loss indicates the signal path energy consumption, d indicates signal transmission distance, the unit is meter, *n* indicates the path-loss factor of the actual environment, f indicates the radio signal power, and the unit is MHz.

The free space propagation path-loss model formula is as follows:

(2) $$P(d)=P(d_{0})+10n\mathrm{lg}(\frac{d}{d_{0}})+\varepsilon ,$$

where P(d) indicates the signal path-loss when the actual measurement distance is d; the unit is dBm. $P(d_{0})$ indicates the signal path-loss when the actual measurement distance is $d_{0}$. The path-loss refers to the absolute power and n indicates the path-loss factor. The loss factor has different values in different environments. ε indicates the shadowing factor, the standard deviation ranges from 4 to 10, and the unit is dB. In this paper, the loss model of distance is $d_{0}=1m$, that is, $\varepsilon \sim N(0,\delta^{2})$.

(3) $$RSSI=P_{t}-P(d),$$

where $P_{t}$ indicates the signal transmitting power; the unit is dBm. P(d) indicates the signal path-loss when the actual measurement distance is d. RSSI indicates the signal strength value when the receiving node distance is $d_{0}$. Using Equations (2) and (3), the following formula can be obtained:

(4) $$RSSI=P(d)-10n\mathrm{lg}(\frac{d}{d_{0}}).$$

From the above formula, when $d_{0}=1m$, the relationship between the intensity and the distance is as follows:

(5) $$d=10^{\frac{P(d)-RSSI}{10n}}.$$

### 3.2. RSSI Data Processing Method

Each node repeatedly measures intensity and then collects a large number of test data as far as possible to remove the error and the noise data. To obtain the optimal intensity data, the chosen intensity data will use the wireless signal loss model to estimate the distance of the nodes.

#### 3.2.1. Experimental Data Acquisition

**Figure 1:**
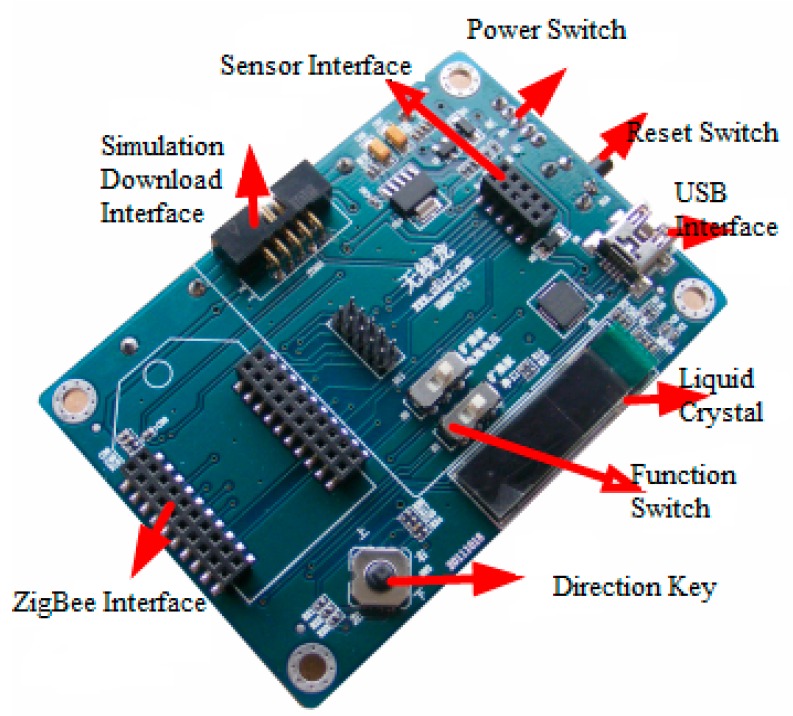
Sensor network node.

The sensor network system used is from the WIRELESS DRAGON TECHNOLOGY COMPANY (Chengdu, China). The node uses the CC2530 module, as shown in Figure 1. Its application development environment uses Microsoft Visual Studio 2010. Data storage and operations use Microsoft SQL Server 2008. The communication protocol uses the ZigBee protocol based on IEEE 802.15.4.

In the experiment, the node receives the signal from the gateway transmission equipment. The node records the signal strength values and sends the data packets to the gateway transmission equipment. Intensity data is from different locations in the gateway node; in the whole process of the experiments, the actual distance is around 25 m from gateway to node. If it exceeds the range, because signal attenuation is too big, the measured RSSI value is not accurate. The application background of node localization has no special value.

The Table 1 shows the strength value of four nodes in different positions. In the experiment, the intensity data is 1000 sets. Statistics are performed on multiple datasets for each measurement node.

#### 3.2.2. Experimental Data Acquisition

The RSSI data can be approximated by a normal distribution. The curve of the peak shows the locations of the optimal RSSI values and the corresponding distance *d* is regarded as the optimal distance. From the experimental data, we can see that the RSSI data are in line with the normal distribution. In this paper the Gauss fitting method is used to select the optimal RSSI value. The Gauss fitting function is as follows:

(6) $$f(x)=a*e^{\frac{-{\left(x-b\right)}^{2}}{c^{2}}},$$

where parameter value $b=\frac{\sum_{i=1}^{n}RSSI_{i}}{n}$, $c=\sqrt{\frac{\sum_{i=1}^{n}\left(RSSI_{i}-b\right)}{n-1}}$.

**Figure 2:**
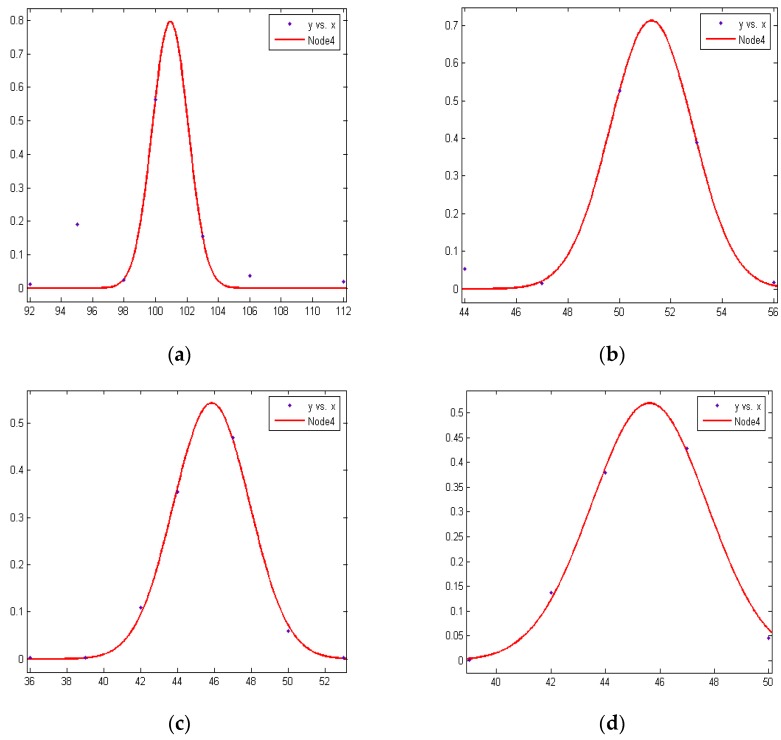
RSSI fitting of Node 4 at (**a**) 1 m; (**b**) 2 m; (**c**) 3 m; and (**d**) 4 m, respectively.

In the above formula, *a* is constant and greater than 0, *b* is the average strength value, and *c* equals the standard deviation. In this paper, we use four nodes to carry out multiple sets of measurements. Figure 2 gives the RSSI fitting curve of Node 4 at 1 m, 2 m, 3 m, and 4 m, respectively, where the abscissa is the RSSI value and the ordinate is the probability value.

#### 3.2.3. Wireless Signal Transmission Loss Model

By obtaining the optimal intensity value, the distance is estimated using the wireless signal loss model. According to $n=\frac{P(d)\;-RSSI}{10\mathrm{lg}(\frac{d}{d_{0}})}$, it can be seen that the environmental factor indicates the degree of loss in the actual transmission, and the numerical value of the signal varies with the change in the distance. Table 2 gives the *n* calculation results when the distance is 1.4 m, 2.2 m, 3.2 m, and 4.5 m.

From the above table, we can get the result of the multi-group *n* value. The average value of each node is obtained, and the formula is as follows:

(7) $$n_{node1}=\frac{\sum_{\left(i=1.4,2.2,3.2,4.5\right)}^{4}n_{i}}{4}.$$

In the formula, $n_{node\;1}$ indicates the environmental factor. The average *n* value is used and the formula is as follows:

(8) $$n=\frac{n_{node1}+n_{node2}+n_{node3}+n_{node4}}{4}.$$

Combining the environmental factor *n*, the wireless transmission loss model, the strength P(d)=103(dBm), and the distance $d_{0}=1m$, the formula is as follows:

(9) $$P(d)=103.1-10*7.773\mathrm{lg}(\frac{d}{d_{0}})-\varepsilon .$$

### 3.3. Region Division Method

A multi-granularity partition method is proposed in this paper. Firstly, according to the characteristics of Thiessen polygons, positioning areas were preliminarily divided using the anchor node location and communication. Secondly, the overlapping portions are acquired by combining the adjacent nodes triangle with a polygon area. By gradually narrowing the scope, we will lock target nodes into the possible regional positioning. Finally, the coordinate is calculated using the localization algorithm.

**Figure 3:**
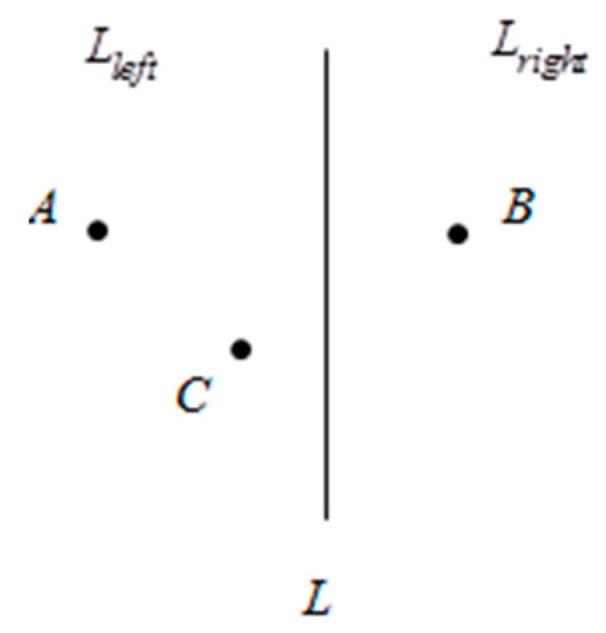
Theorem description graph.

**Definition** **1.** *Take a plane with two different points* A *and* B*.* C *is the perpendicular bisector of the line from* A *to* B*. The plane is divided into two parts:* $L_{right}$ *and* $L_{left}$*, where* $A\in L_{left}$*,* ∃P*,* |PA|<|PB|*, that is, the point-to-point distance from* P *to* A *is less than the point-to-point distance from* P *to* B*, namely the space located inside the plane* $L_{left}$ *is closer to point* P*, as shown in Figure 3.*

**Definition** **2.** *Take a plane with* N *different points* $\left\{P_{1},P_{2},...,P_{n}\right\}$*. For any point* $P_{i}$*, there is a closest point distance* $P_{2}$*, and the distance from* $P_{2}$ *to* $P_{i}$ *is less than the distance from* $P_{2}$ *to other points, that is, the region* $V_{p}$ *contains* $P_{i}$ *in the intersection of* N−1 *half plane. The* N−1 *half plane is determined by the perpendicular bisector of* $P_{i}$ *and other points, where region* $V_{p}$ *is composed of a plurality of vertical bisectors of the polygon.*

By the above definition, the plane is divided into N regions $V_{P_{i}}$; each region $V_{p}$ has a center, and the edges of $V_{p}$ are two adjacent lines of a perpendicular bisector. These two points are known Thiessen polygons, with the edges of $V_{p}$ between the focus and vertex. To sum up: if $(x,y)\in V_{P_{i}}$, $P_{i}$ is the closest point of the distance from (x,y).

#### 3.3.1. Regional Primary Division

**Figure 4:**
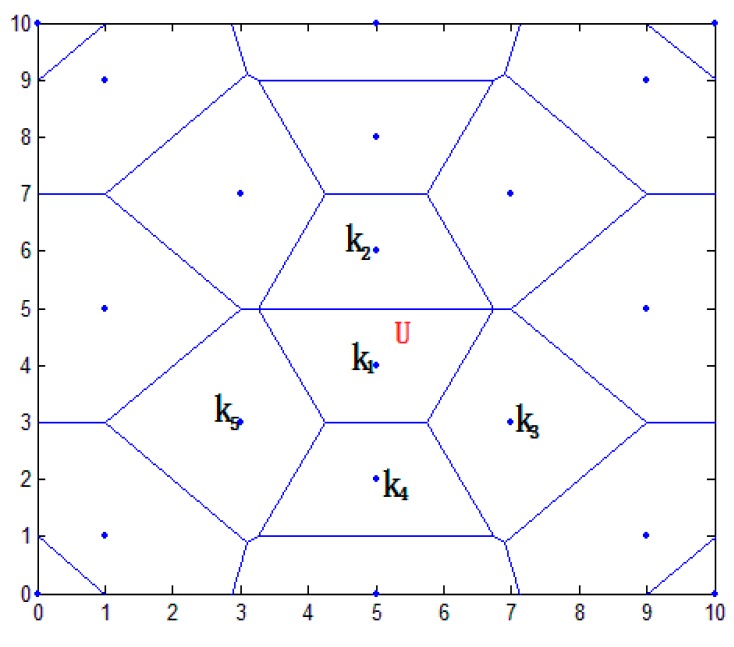
Overall positioning results.

In this paper, the distance vector is established. According to the characteristics of Thiessen polygons, the area was preliminarily divided to narrow it down. In Figure 4, this paper establishes an overall positioning area of $10\times 10\;m^{2}$, placing a plurality of anchor nodes. The blue point shows the position of anchor nodes; the blue line shows the perpendicular bisector of the anchor line. The closed region is a polygon using the anchor node as the center. Because each node belongs to the respective area, the whole positioning area is divided into a plurality of smaller areas. In the node communication radius, the target node U communicates with anchor nodes $K_{1},K_{2},...,K_{n}$. Sequence distance $d_{1},d_{2},...,d_{n}$ are acquired between the target node U and anchor node $K_{1},K_{2},...,K_{n}$.

In Figure 4, the nearest anchor node $K_{1}$ of the target node U is calculated, and the sequence distance $\left\{d_{k_{2}},d_{k_{3}},...,d_{k_{s}}\right\}$ between $K_{1}$ and the adjacent nodes $K_{2},K_{3},...,K_{s}$ is calculated (i≤6). The formula is as follows:

(10) $$d=\sqrt{{\left(x_{1}-x_{2}\right)}^{2}+{\left(y_{1}-y_{2}\right)}^{2}},$$

where $\left(x_{1},y_{1}\right)$ and $\left(x_{2},y_{2}\right)$ are the respective coordinates of the anchor nodes. d is the distance between $K_{1}$ and $K_{2}$. The vertical line of the connecting line from $K_{1}$ to $K_{2},K_{3},...,K_{s}$ is established, and a closed area is composed of a plurality of vertical lines. The area is a polygonal area using an anchor node $K_{1}$ as the center, called $V_{K_{1}}$.

To determine U: if it is in $V_{K_{1}}$, the area will continue division; if it is not in $V_{K_{1}}$, it will select sequence distance ranking second anchor node $K_{2}$. If U is within $V_{K_{2}}$, it is found one $V_{K_{\;i}}$, which the target node U is in this region $V_{K_{\;i}}$. According to the nature of the Thiessen polygon that can be drawn, if the target node U is within this region $V_{K_{1}}$, it needs to meet the following conditions, where $d_{\left(U,K_{1}\right)}$ is the distance from U to $K_{1}$, $d_{\left(U,K_{1}\right)}$ is the distance between U and $K_{1},K_{2},...,K_{n}$:

(11) $$V_{K_{1}}=\left\{K|d_{\left(U,K_{1}\right)}\leq d_{\left(U,K_{j}\right)},j=2,3....n\right\}.$$

#### 3.3.2. Region Dividing Based on Second Division

**Figure 5:**
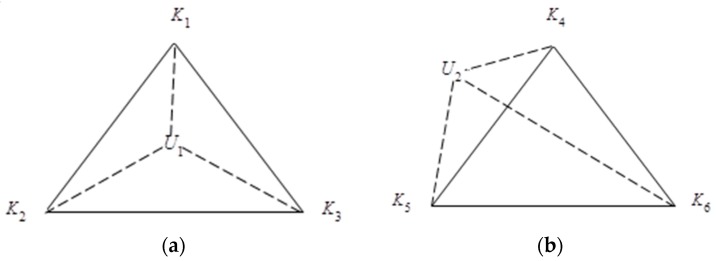
The target node being (**a**) inside and (**b**) outside the triangle area.

After dividing, the polygon area is still large, so a sequence table is established to choose the anchor node closest to the unknown node. At this time, the unknown node region is wide, and the final judgment coordinate sequences are not entirely accurate. Therefore, in this paper, we consider overlapping region of polygons and triangles. As far as possible we will target the minimum range, and then calculate the coordinates of the target node. The main division process is as follows:

According to the region division it has been judged that U is in $V_{K_{1}}$. At the same time, it can get the sequence distance $d_{1},d_{2},...,d_{n}$ between U and $K_{2},K_{3},...,K_{5}$. The distance sequence $d_{K_{2}},d_{K_{3}},...,d_{K_{5}}$ between $K_{1}$ and $K_{2},K_{3},...,K_{5}$. $K_{1}$, $K_{2},K_{3},...,K_{5}$ is composed of a number of triangles, where $K_{1}$ will be regarded as a fixed point in the triangle, and the rest of the vertices are $K_{2},K_{3},...,K_{5}$. The probability of target node U being in $\Delta K_{1}K_{2}K_{3}$ is assessed according to the following description:

In Figure 5, $U_{1}$ and $U_{2}$ are the target node, $U_{1}$ is in the triangle area $\Delta K_{1}K_{2}K_{3}$, and $U_{2}$ is outside the triangle area $\Delta K_{4}K_{5}K_{6}$. To determine whether a point is in the triangle, two common methods are used: area method and vector directionmethod. In this paper, the method of area is used to judge whether the target node is in the triangle, and the formula is as follows:

(12) $$S_{ABP}+S_{BCP}+S_{ACP}\leq S_{ABC}.$$

**Figure 6:**
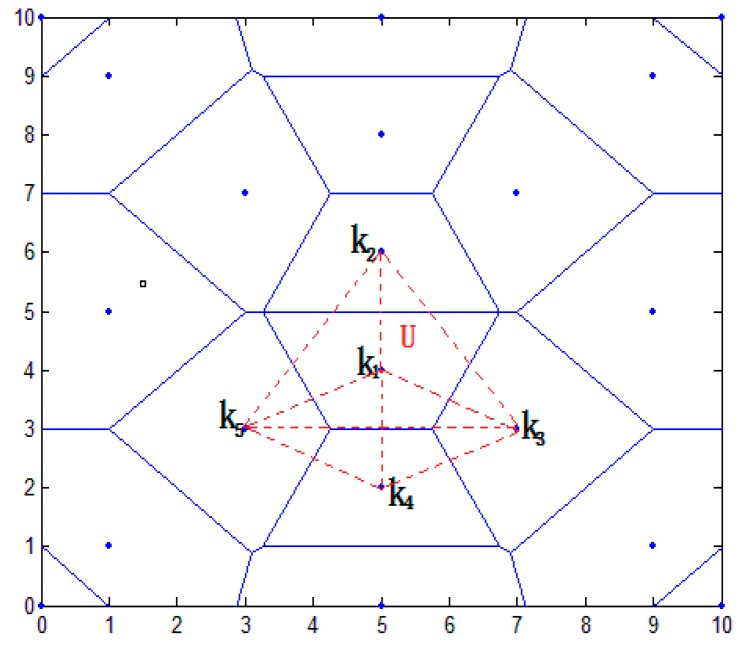
Results of triangle localization.

In Figure 6, the red dotted line is composed of triangles, while the area is represented by the continued division in Figure 5. Because each polygon area can be divided into a number of triangular regions, they can be overlapping.

#### 3.3.3. Analysis of Regional Division Method

The region partition process includes main two parts. Firstly, the whole region is divided into a plurality of sub-regions using the Thiessen polygon method. Secondly, a combination of adjacent nodes forms a triangular region. Lastly comes the judgment of the final area for unknown nodes. The area partition method pseudo codes (Algorithm 1) are as follows:
  **Algorithm 1**: LocationRegional(N)  Input: A set of all anchor nodes: $N=\{N_{1},N_{2}...,N_{n}\}$    j unknown nodes evenly distributed in the location region;  Output: Localization algorithm for unknown node P  1. A set of anchor nodes N marked serial number for n anchor nodes  2. FOR (i = 1 to j)  3. RUN P_RSSI(i), obtaining the strength vector set between N and P $P\_RSSI=\{R_{1},R_{2},...,R_{n}\}$.  4.   P_D(i), obtaining the strength vector set between N and P $P\_D=\{D_{1},D_{2},...,D_{n}\}$  5.   P_d(i), sorting P_D(i) in ascending order  6.   P_d(i)_top1, taking the first anchor node O  7. FOR (i = 1 to n-1)  8.   RUN O_rssi(i), obtaining the strength vector set between O and the other anchor node, $O\_rssi=\{r_{2},r_{3},...,r_{n}\}$.  9.    O_D(i), obtaining the distance vector set between O and the other anchor node, $O\_D=\{D_{1},D_{2},...,D_{n}\}$.  10.    O_d(i), sorting O_D(i) in ascending order  11.    O_d(i)_top 6, taking the top six anchor nodes A, B, C, D, E, and F, using the vertical line with the O midline forming a closed polygon area $V_{O}$.  12.   IF $P\_d_{O}<P\_d_{k}(k=A,B,C,D,E,F)$, judge whether the distance between P and O is less than the other adjacent anchor nodes, O, B, C, D, E, and F.  13.    RUN the $V_{O}$ neighboring anchor nodes A, B, C, D, E, and F following the order of O_d(i)_top 6 forming a number of triangles ΔOij(i,j=A,B,C,D,E,F).  14.     IF $S_{POA}+S_{POB}+S_{PAB}<S_{OAB}$,  15.      RUN InternMethod, selecting internal unit positioning algorithm  16.    ESLE RUN ExternMethod, selecting external unit positioning algorithm  17.   ENDIF  18. ENDFOR  19. ENDFOR

## 4. Node Localization Algorithm Based on Lagrange Multiplication and Taylor Formula

This paper uses a Shadowing Model to show the relationship between the signal intensity and the distance. It uses the Gaussian fitting processing intensity data, and acquires the distance. Firstly, the distance sequence is introduced to select the nearest anchor node. According to the characterization of the Thiessen polygon, the anchor nodes are divided into polygonal regions. Secondly, the neighboring nodes are chosen around anchor nodes. The polygon region division is composed of the triangle using multiple adjacent nodes, so as to determine the unknown node positioning area. Thirdly, by calculating the distance from the virtual reference point to the anchor nodes, the distance vector sequence from reference point to anchor node is established. According to the characteristics of vector similarity, several recent reference points are chosen. Finally, the centroid algorithm is used to calculate the coordinates of unknown nodes. On the other hand, considering the location of edge nodes, if unknown nodes are not in the overlapping region, a close reference point is chosen using the characteristics of vector similarity. Furthermore, the coordinates of the initial node are used in the Lagrange multiplication [24] and Taylor’s formula [25]. By iterative obtainedcoordinates, it may acquire rational coordinates of the unknown node.

### 4.1. Node Location Method in Positioning Unit

The localization idea is acquiring location by dividing the overall region. This can improve the nodes in the edge region, namely non-overlapping regions. Nodes in the interior of the unit are as follows. Firstly, according to the nature of the Thiessen polygon, the unknown node is within this area. Finally, the appropriate virtual reference point is selected to compute the coordinates of the target node.

**Figure 7:**
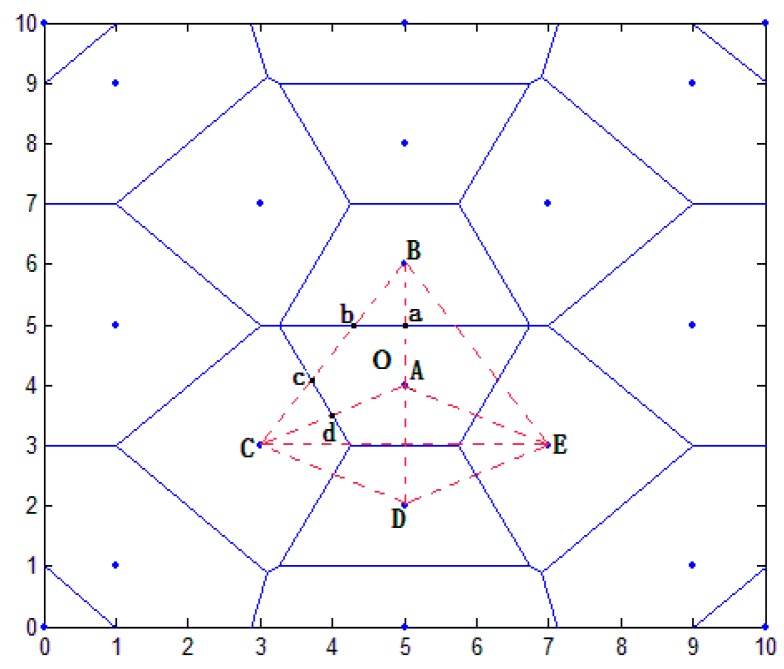
Unknown node in the positioning unit.

According to Figure 7, the location of the unknown node in the unit is as follows:

In the location area, distance *d* is estimated by RSSI. Furthermore *d* is given in ascending order and the ranked the first anchor node must be selected. According to the Thiessen polygon theorem, the polygonal regions of the anchor node are divided. As shown in Figure 7, O represents the unknown node. A, B, C, D, and E represent the anchor nodes. The distance sequence of O, from small to large, is: A→B→C→D→E.

To determine whether the unknown nodes are in the polygon, the specific method is as follows. If the unknown nodes are judged to be within this region, complete the following steps. Otherwise, ranked the second anchor node is selected to judge whether or not the unknown nodes are in this triangle. As shown in Figure 7, O is in the region using A as the center, that is, within $V_{A}$.

Coordinates are acquired in the polygon area. The d sequence between the unknown nodes and the nearby anchor node is acquired. The d values are in ascending order. The number is ranked from 1 to 7 (i≤6). The ranked the first anchor node of d is acquired. According to the *d* sequence, the other anchor nodes form multiple triangles. As shown in Figure 7, O is in $V_{A}$, and neighboring nodes of $V_{A}$ are *B*, *C*, *D*, and *E*; thus, in accordance with the principle of distance sequence, various triangular combinations exist: ΔABC, ΔABD, ΔABE, ΔACD, ΔACE, and ΔADE.

The unknown nodes are in the triangle area. By calculating the distance from each virtual reference node to the anchor nodes, corresponding distance vectors are formed. As shown in Figure 7, O is in VA and in ΔABC (where a, b, c, and d are virtual reference nodes). These reference points are pairwise linear points or points of intersection. Their coordinates can be obtained.

According to the distance vector similarity, we can select 3 the highest similar degree virtual reference node. As shown in Figure 7, the midpoint may be obtained by calculating from the virtual reference nodes a, b, c, and d to anchor node A and then finding the area of the overlap. Finally, the coordinates of unknown nodes are calculated by a centroid algorithm.

### 4.2. Node Location Method outside Positioning Unit

#### 4.2.1. Determining the Location Area of Node

**Figure 8:**
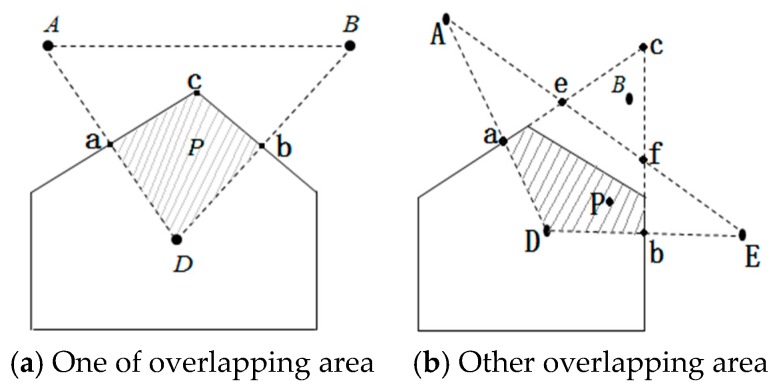
Determining two overlapping area.

As shown in Figure 8a, *P* represents the unknown node. *A*, *B*, and *D* represent three anchor nodes. The polygon region within the solid lines represents a polygon region with *D* as the center, called $V_{D}$. The dotted area represents ΔABD. The shaded area represents the overlap of two regions, $V_{D}$ and ΔABD. *a* and *b* represent the midpoint from *D* to the neighboring nodes *A* and *B*, respectively. *C* represents the intersection point. $V_{D}$ is intersected by two edges, forming a solid line area. The *A*, *B*, and *D* coordinates are known: $\left(x_{A},y_{A}\right)$, $\left(x_{B},y_{B}\right)$, and $\left(x_{D},y_{D}\right)$. If *a* and *b* are the midpoints, you can get the virtual reference point a, b coordinates; the formula is as follows:

(13) $$(x_{a},y_{a})=\left(\frac{x_{D}+x_{A}}{2},\frac{y_{D}+y_{A}}{2}\right).$$

Also, the intersection C is line $L_{DA}$ and $L_{DB}$ perpendicular.

As shown in Figure 8b, *P* represents the unknown node. *A*, *B*, *D*, and *E* represent four anchor nodes. The area within the solid lines represents a polygon region with D as the center, called $V_{D}$. The dotted area represents ΔADE. The shading represents region $V_{D}$ and ΔADE in the overlapping region. The distance sequence from *P* to *A*, *B*, *D*, and *E* is ordered from small to large as follows: *D*→*E*→*A*→*B*, thus judging that *P* is in ΔADE, where *a* and *b* represent the midpoint between *D* and *A* or *E*, respectively. *C* represents the intersection by the perpendicular bisector of the line through $L_{DA}$ and $L_{DB}$.

The overlap region does not select coordinates of the intersection, as shown in Figure 8b. *e* represents line $L{\;}_{AE}$ and $L_{DA}$ the perpendicular bisector intersection. *f* represents line $L{\;}_{AE}$ and $L_{DA}$ the perpendicular bisector intersection. Selecting intersections *e* and *f* determines the overlapping region more accurately. It may guarantee a point *P* being in ΔADE. The coordinates of the intersection are as follows:

(14) $$x=\frac{b_{ae}-b_{AE}}{k_{AE}-k_{ae}},y=k_{AE}*\frac{b_{ae}-b_{AE}}{k_{AE}-k_{ae}}+b_{AE},$$

where the values of the parameters are, respectively, $b_{ae}=\frac{\left(x_{a}y_{a}+1)(y_{A}-y_{D}\right)}{x_{a}(x_{A}-x_{D})}$, $k_{ae}=\frac{x_{D}-x_{A}}{y_{A}-y_{D}}$, $b_{AE}=y_{A}-\frac{x_{A}(y_{A}-y_{E})}{x_{A}-x_{E}}$, and $k_{AE}=\frac{y_{A}-y_{E}}{x_{A}-x_{E}}$.

**Figure 9:**
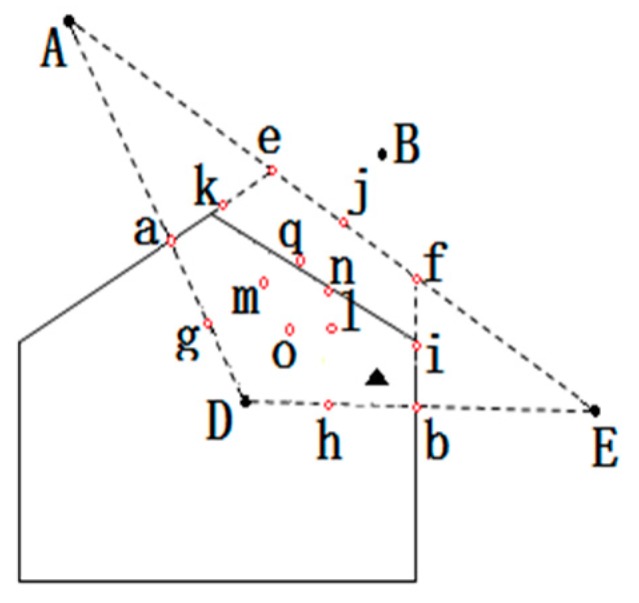
Virtual reference points.

Some virtual reference points can be obtained for the overlapping area. The coordinates of the reference points are for the midpoint Equation (13) obtained, as shown in Figure 9. Anchor nodes are *A*, *D*, and *E*; virtual reference point h is the midpoint between *D* and *B*. The small red circles are virtual reference points and the black triangle represents the unknown node.

#### 4.2.2. Selecting Virtual Reference Node

There is an unknown node *P* whose coordinates are (x,y); reference nodes *P* are *A*, *B*, and *C*, and their coordinates are (0,0), (1,1), (0,2), respectively. *P* is in ΔABC; the distance can be obtained by the following equation:

(15) $$\left\{ \begin{array}{l} {d_{AP}}^{2}={\left(0-x\right)}^{2}+{\left(0-y\right)}^{2}\\ {d_{BP}}^{2}={\left(1-x\right)}^{2}+{\left(1-y\right)}^{2}\\ {d_{CP}}^{2}={\left(0-x\right)}^{2}+{\left(2-y\right)}^{2} \end{array}\right..$$

In the above equation group, the coordinates (x,y) are judged by the distance d.

**Lemma** **1.** *In the positioning unit, there are a number of unknown nodes* $P_{i}(i=0,1,2,...,n)$*. The distance vector between any P_i_ and the reference node is corresponding to the coordinates of that point, moreover the value of the coordinates is only one.*

**Definition** **3.** *In the regional location, there is a sample point, denoted as* $x_{i}$*. The sample point can receive the other node strength RSSI value and form a RSSI vector set sorted in descending order, i.e.,* $R_{1}=\{r_{11},r_{12},...,r_{1n}\}$*, where* $r_{1}$ *denotes the sample* $x_{1}$ *collecting the strength value from node 1 and* $R_{1}$ *denotes the sample* $x_{1}$ *collecting the strength value of all nodes.*

**Definition** **4.** *In the location area, there are multiple samples* $x_{1},x_{2},...,x_{n}$*, and each sample point can receive the other node strength RSSI value. From the many groups an RSSI vector set is formed and sorted in descending order, i.e.,* $R=\{R_{1},R_{2},...,R_{n}\}$*, where* $R_{1}$ *represents samples* $x_{1}$ *collecting the intensity values from all the nodes and R represents the samples* $x_{1},x_{2},...,x_{n}$ *collecting the strength set from all nodes.*

According to Definitions 3 and 4, the RSSI vector set is as follows:

(16) $$R=\left[\begin{array}{c} R_{1}\\ R_{2}\\ .\\ .\\ R_{k} \end{array}\right]=\left[\begin{array}{cccc} r_{11} & . & . & r_{1n}\\ r_{21} & . & . & r_{2n}\\ . & . & . & .\\ . & . & . & .\\ r_{k1} & . & . & r_{kn} \end{array}\right].$$

According to Equation (16), we can get the corresponding distance vector set:

(17) $$D=\left[\begin{array}{c} D_{1}\\ D_{2}\\ .\\ .\\ D_{k} \end{array}\right]=\left[\begin{array}{cccc} d_{11} & . & . & d_{1n}\\ d_{21} & . & . & d_{2n}\\ . & . & . & .\\ . & . & . & .\\ d_{k1} & . & . & d_{kn} \end{array}\right],$$

where $d_{11}$ represents the distance vector between the sample point $x_{1}$ and the node 1; $D_{1}$ represents the distance vector of the sample points $x_{1}$ between and all nodes; and D represents the distance vector set from all the sample points $x_{1},x_{2},...,x_{n}$ to all nodes.

The distance from each unknown node to the anchor nodes produces a set of vectors. Using fuzzy mathematics, we can examine the close degree of unknown nodes, called vector similarity. In this paper, we establish the distance vector between the unknown node and the anchor node, and the distance vector between the reference node and the anchor node. The vector cosine similarity [26] is used to select the nearest reference node.

Vector cosine similarity is used to measure the degree of individual differences; the parameter is the cosine of the angle between the two vectors. The formula is as follows:

(18) $$sim(X,Y)=\cos\theta =\frac{\vec{x}*\vec{y}}{\left\|x\right\|*\left\|y\right\|}.$$

If *A* and *B* are two *n*-dimensional vectors, *A* is $\left[A_{1},A_{2},...,A_{n}\right]$, *B* is $\left[B_{1},B_{2},...,B_{n}\right]$, the cosine value of angle θ between *A* and *B* is calculated as follows:

(19) $$\cos\theta =\frac{\sum_{i=1}^{n}\left(A_{i}*B_{i}\right)}{\sqrt{\sum_{i=1}^{n}{\left(A_{i}\right)}^{2}}*\sqrt{\sum_{i=1}^{n}{\left(B_{i}\right)}^{2}}}=\frac{A*B}{\left|A|*|B\right|}.$$

**Figure 10:**
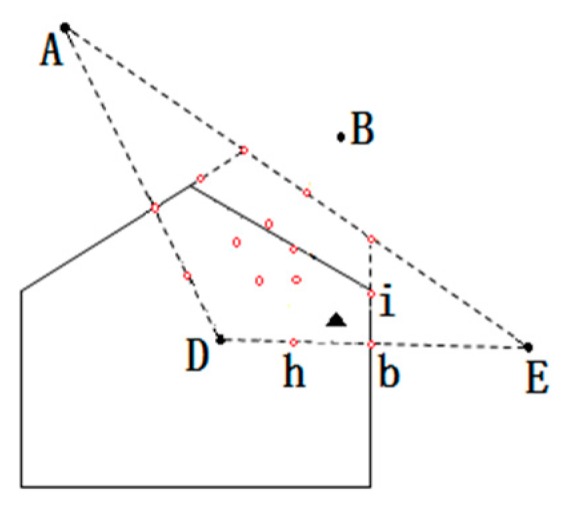
Closest virtual reference nodes.

As shown in Figure 10, *A*, *B*, *D*, and *E* represent anchor nodes, and the black triangle represents unknown node *P*. At the same time, it has been determined that *P* is in $V_{D}$ and also in ΔDAE. Next the virtual reference point is acquired. A red point represents a virtual reference point; reference points *h*, *b*, and *i* are close to the virtual reference point.

The main selection process is as follows. Firstly, *P* collects the RSSI from *A*, *D*, and *E* and forms the vector set in descending order. Then it may estimate each RSSI value corresponding to the distance value *d* using a wireless signal loss model. This can also form a distance vector $\left\{d_{PD},d_{PE},d_{PB}\right\}$ using *A*, *D*, and *E*. Secondly, in selected areas we can compute the distance from all virtual reference points to *A*, *D*, and *E*. Using the Euclidean distance formula we can obtain each virtual reference point in the *A*, *D*, *E* distance vector, called $\left\{d_{iD},d_{iE},d_{iB}\right\}$. Before the distance vector is formed, each distance value is sorted according to the corresponding distance. In this paper, the rules for anchor nodes are sorted according to the distance, where $d_{iD}$ represents the distance between virtual reference point *i* and anchor node *D*. Lastly, the distance vector sequence similarity is used to compute the similarity degree between $\left\{d_{PD},d_{PE},d_{PB}\right\}$ and $\left\{d_{iD},d_{iE},d_{iB}\right\}$. The formula is as follows:

(20) $$\cos\theta =\frac{d_{PD}*d_{iD}+d_{PE}*d_{iE}+d_{PA}*d_{iA}}{\sqrt{{d_{PD}}^{2}+{d_{PE}}^{2}+{d_{PA}}^{2}}*\sqrt{{d_{iD}}^{2}+{d_{iE}}^{2}+{d_{iA}}^{2}}}.$$

The cosθ value of the formula is close to 1, which indicates that the angle is close to 0, that is, the two vectors are more similar. The distance similarity between virtual reference nodes and unknown nodes are computed. Furthermore, similarity is given in descending order and the virtual reference point is used when ranking the top three. The *P* distance vector and *h*, *b*, *i* distance vector are similar, that is, *h*, *b*, *i* was close to *P*’s three virtual reference nodes.

Finally, the centroid algorithm is used to calculate the coordinates of unknown nodes *P*. Figure 8 is an example and the formula is as follows:

(21) $$(x,y)=\left(\frac{x_{h}+x_{b}+x_{i}}{3},\frac{y_{h}+y_{b}+y_{i}}{3}\right).$$

#### 4.2.3. Determining the Range of Node Coordinates

**Figure 11:**
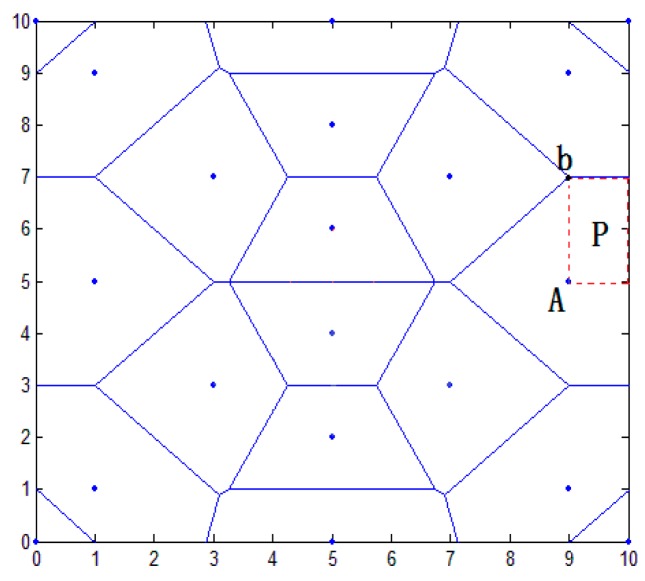
Determining the range of unknown node coordinates.

As shown in Figure 11, *P* represents the unknown node, *A* represents the nearest anchor node from the *P*, and *P* is in the region $V_{A}$. B represents the nearest virtual reference point to P.

The steps for determining the coordinate range are as follows:

Step 1. According to the scope of the regional positioning, the boundary equation is acquired, as well as the computing distance from the center of the polygon region to the boundary. As in Figure 11, the boundary of location area are x=0,x=10,y=0 and y=10, respectively, and we can calculate the distance from A to x=0,x=10,y=0 and y=10 in ascending rank order.
Step 2. By choosing the distance value, we can get the distance of the corresponding boundary equations, and the value of this equation is considered as one of the candidate coordinates. For example, the minimum distance value $d_{A2}$, which represents the distance between A and x=10. Then x=10 will be considered as a candidate.
Step 3. Taking A and B coordinate values and selecting candidate values using the above step, all values are compared, such as $A=(x_{A},y_{A})$, $b=(x_{b},y_{b})$, x=10. There are three coordinates being compared, i.e., $x_{A},x_{b},x$. The comparative value of y are two, i.e., $y_{A},y_{b}$. The number of comparative value is determined by candidate value.
Step 4. Given the comparative values of x,y, and the $x_{\min}=x_{A},x_{\max}=10,y_{\min}=x_{A},y_{\min}=x_{b}$, the final coordinates are determined, that is, $\left(x_{\min}\leq x\leq x_{\max},y_{\min}\leq y\leq y_{\max}\right)$.

#### 4.2.4. Regional Primary Division

In this paper, given the two-variables function $f(x,y)={\left(x_{i}-x\right)}^{2}+{\left(y_{i}-y\right)}^{2}-d_{i}^{2}$, the minimum value is computed, namely, the minimal difference between the actual distance and the calculated distance.

**Figure 12:**
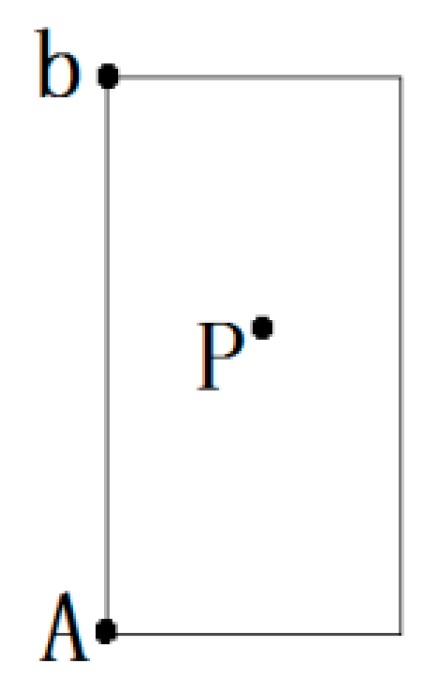
Unknown node coordinates range.

As shown in Figure 12, *P* represents the unknown node, *A* represents the nearest anchor node to *B*, and *b* represents the virtual reference point. The rectangular region represents *P*’s location region, that is, the coordinate range of *P*. The calculated coordinate process is shown as follows:

Given the two-variables function $f(x,y)=\left|{\left(x_{i}-x\right)}^{2}+{\left(y_{i}-y\right)}^{2}-d_{i}^{2}\right|$, the minimum value f(x,y) is computed, namely, the minimal difference between ${\left(x_{i}-x\right)}^{2}+{\left(y_{i}-y\right)}^{2}$ and $d_{i}^{2}$. In order to simplify the calculations, this paper only discusses the extreme value of the function f(x,y)≥0, that is, ${\left(x_{i}-x\right)}^{2}+{\left(y_{i}-y\right)}^{2}\geq d^{2}$; the formula is as follows:

(22) $$f(x,y)={\left(x_{A}-x\right)}^{2}+{\left(y_{A}-y\right)}^{2}-{d_{A}}^{2}.$$

In the above formula, $\left(x_{A},y_{A}\right)$ are the nearest anchor node coordinates from unknown nodes; the distance between *A* and *P* is expressed as $d_{A}^{2}={\left(x_{i}-x\right)}^{2}+{\left(y_{i}-y\right)}^{2}$; and (x,y) are the unknown node’s coordinates.

The node coordinate calculation model is established by the Lagrange multiplier method as follows:

(23) L(x,y)=f(x,y)+λ*φ(x,y).

In the above formula, f(x,y) represents the two-variables function about anchor node coordinates, unknown node coordinate, measuring distance; φ(x,y) represents the unknown node area, namely node coordinate constraint condition. L(x,y) represents the positioning function model and λ represents the parameter. By the constraint of node coordinates $\left(x_{\min}\leq x\leq x_{\max},y_{\min}\leq y\leq y_{\max}\right)$, the constraint equation is established as follows:

(24) φ(x,y)=x+y−ε,

where x,y represents the coordinates of the unknown nodes, and ε represents the sum of the range (x,y), that is, $\varepsilon =(x_{\max}-x_{\min})+(y_{\max}-y_{\min})$. Using the Lagrange multiplier method, the general equation is obtained as follows:

(25) $$L(x,y)={\left(x_{A}-x\right)}^{2}+{\left(y_{A}-y\right)}^{2}-{d_{A}}^{2}+\lambda *(x+y-\varepsilon ).$$

According to the above Equations (24) and (25), the partial derivative of *x*, *y* is computed respectively. Let the partial derivative equal zero and combine the constraint Equation (24) establishing a set of equations, as follows:

(26) $$\left\{ \begin{array}{l} L^{\prime }x(x,y)=f^{\prime }x(x,y)+\lambda *\varphi^{\prime }x(x,y)=0\\ L^{\prime }y(x,y)=f^{\prime }y(x,y)+\lambda *\varphi^{\prime }y(x,y)=0\\ \varphi (x,y)=0 \end{array}\right..$$

By solving the equations, we can get the value x,y,λ. At this time x,y are the extreme point coordinates, that is, the extreme points in the function z=f(x,y) in constrained conditions. The above figure is an example, using the Lagrange multiplier method; the full equations are as follows:

(27) $$\left\{ \begin{array}{l} 2x-2x_{A}+\lambda =0\\ 2y-2y_{A}+\lambda =0\\ x+y-\lambda =0 \end{array}\right..$$

Solving the above equations, x,y,λ are acquired.

Firstly, by using the method of Lagrange, the initial coordinates (x,y) are obtained; $\left(x_{n},y_{n}\right)$ indicates the coordinates of the anchor nodes. f(x,y) is determined as follows:

(28) $$f(x,y)=\sqrt{{\left(x_{n}-x\right)}^{2}+{\left(y_{n}-y\right)}^{2}}.$$

The formula is expanded in the (x,y) Taylor series; the modifying step size *k* and only the first-order partial derivative are retained, and the higher order functions are ignored:

(29) $$\begin{array}{l} f(x+h,y+k)=f(x,y)+(h\frac{\partial }{\partial x}+k\frac{\partial }{\partial y})f(x,y)\\ =f(x,y)+h\frac{\left(x-x_{n}\right)}{f(x,y)}+k\frac{\left(y-y_{n}\right)}{f(x,y)} \end{array}.$$

From the above calculations, the coordinates of the range are $\left(x_{\min}\leq x\leq x_{\max},y_{\min}\leq y\leq y_{\max}\right)$. The fixed step size *k*, *h* range is determined, that is, $\left(x_{\min}-x\leq h\leq x_{\max}-x,y_{\min}-y\leq k\leq y_{\max}-y\right)$, and then the threshold is acquired, $\sqrt{h^{2}+k^{2}}<\alpha$. Combining the two equations above, calculation step correction value, and judgment *h*, *k* meets the threshold. If it meets the threshold, the current coordinates are the final coordinates of the unknown nodes. If it does not satisfy the threshold, iteration step size *h*, *k* is modified until it meets the threshold, finally returning to the current coordinates.

## 5. Localization Algorithm Simulation and Experiment

### 5.1. Simulation Results

Anchor nodes provide different coverage and thus different simulation environments are established. The experimental platform is MATLAB 7.0 and MyEclipse 9.0. First of all, a $10\times 10\;m^{2}$ square area simulation environment is established. The anchor node number is 8, 16, 24, or 32. Unknown nodes are evenly distributed throughout the whole positioning region. Assume that all unknown nodes can communicate with the anchor nodes.

**Figure 13:**
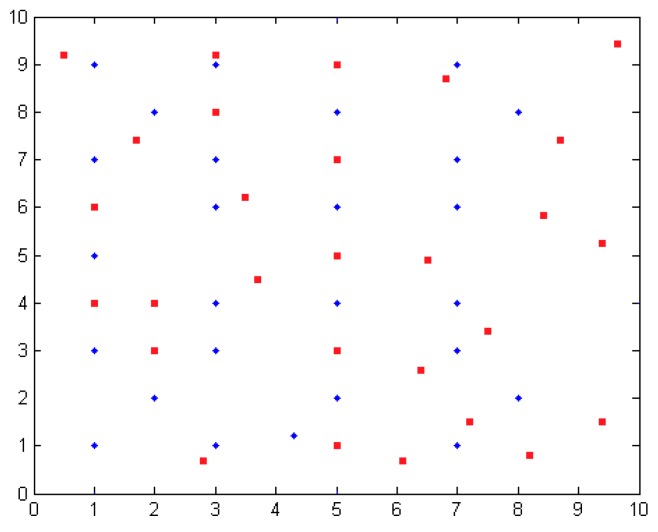
One of the simulation location area distribution maps.

**Figure 14:**
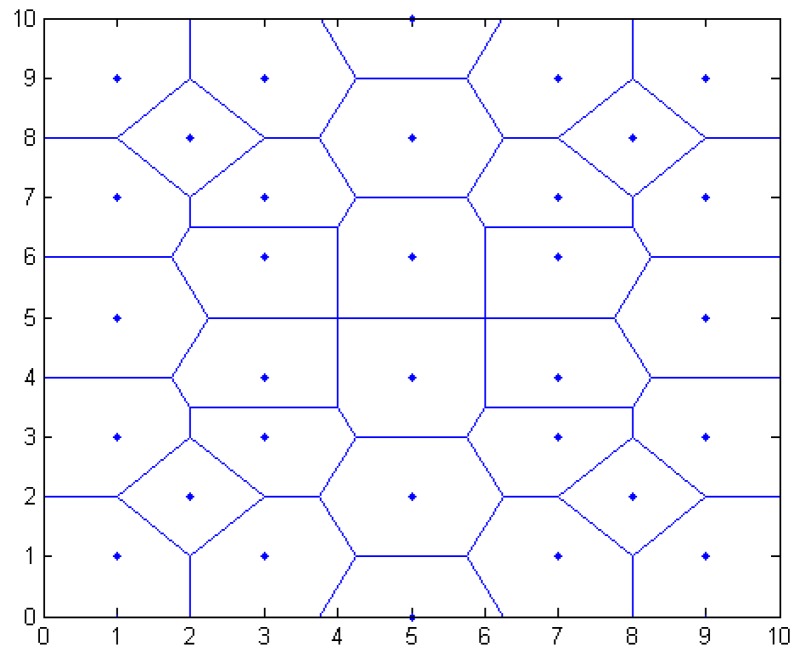
Location division map.

As shown in Figure 13, the red dot represents a randomly generated unknown node; the blue point represents the anchor node coordinates. Taking the number of anchor nodes to be 32 for an example, shown in Figure 14, the regional positioning is as below, where a blue point represents a location within all anchor nodes and solid lines enclose a polygon region.

**Figure 15:**
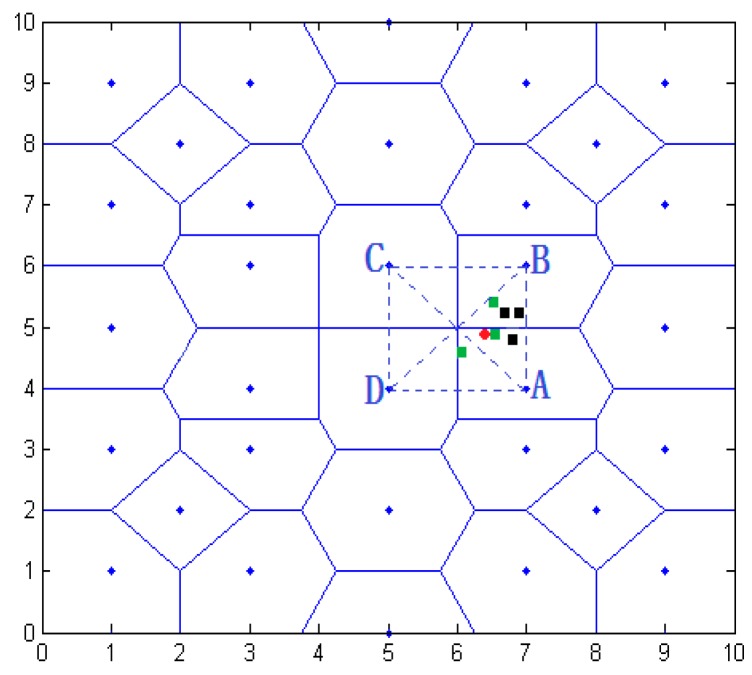
Region division positioning result.

This paper uses original strength value fluctuation of 5% and 10% in simulating. Taking the original coordinates of unknown nodes (6.5, 4.9) as an example, shown in Figure 15, the blue represents all the anchor nodes in the location area. Solid lines represent each anchor node in the center partition polygon area, in which a, b, c, and d represent anchor nodes with respective coordinates of (7.0, 4.0), (7.0, 6.0), (5.0, 6.0), and (5.0, 4.0). The dotted lines form a four-triangle area. The small red dot represents the original position of the unknown nodes, with coordinates of (6.5, 4.9), The black squares represent intensity fluctuation, being 5% at the estimated coordinates. Green dots represent the estimated node coordinates, when the intensity fluctuation of 10%. With intensity fluctuations, the node localization results are Table 3 and Table 4.

In this paper, the error formula is as follows:

(30) $$e=\frac{\left|\left(x_{0},y_{0})-(x,y\right)\right|}{R}\times 100\%,$$

where $\left(x_{0},y_{0}\right)$ are the coordinates, the actual measurement coordinates is (x,y), and the communication radius is R.

**Figure 16:**
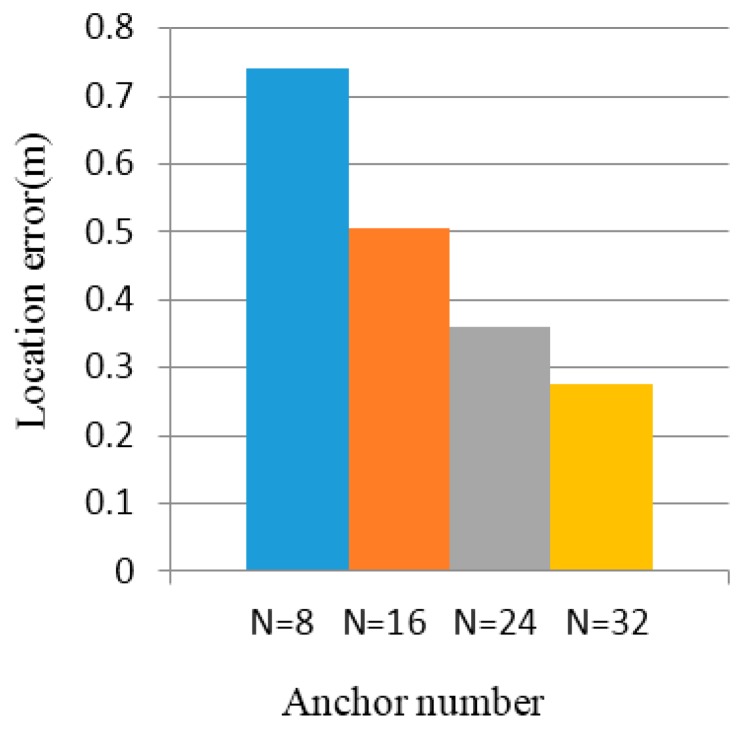
Location error when intensity value is fluctuation 5%.

**Figure 17:**
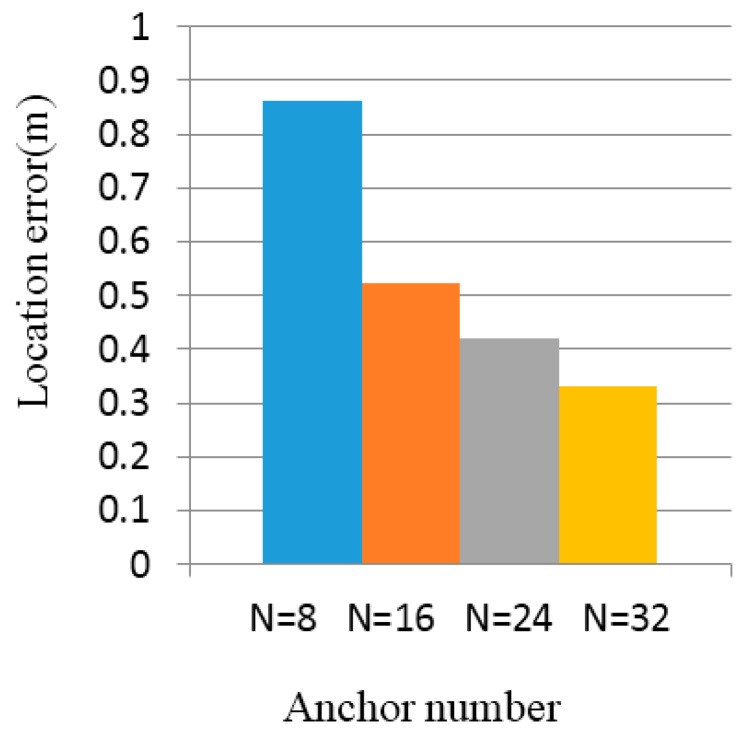
Location error when intensity value is fluctuation 10%.

As shown in Figure 16 and Figure 17, the abscissae of the two charts reflect the number of anchor nodes: N=8,N=16,N=24,N=32, respectively.

**Figure 18:**
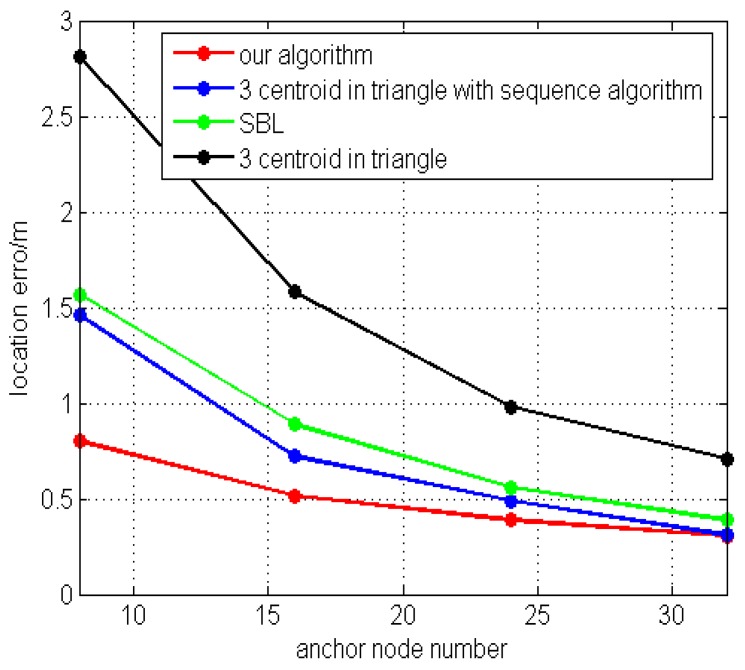
Comparing location algorithm error.

Figure 18 shows the localization algorithm, SBL [18], three-centroids in a triangle with a sequence algorithm [19], and three-centroids in a triangle [14]; the error trends are correlated with the number of anchor nodes. The position error curve basically showed a decline. More than 20 unknown nodes are randomly generated, and calculate the coordinates of these nodes, when the number of anchor nodes are N=8,N=16,N=24,N=32, respectively; the average positioning error of the unknown nodes are 0.8 m, 0.5 m, 0.4 m, and 0.3 m. The positioning error of the algorithm is less than 1 m.

**Figure 19:**
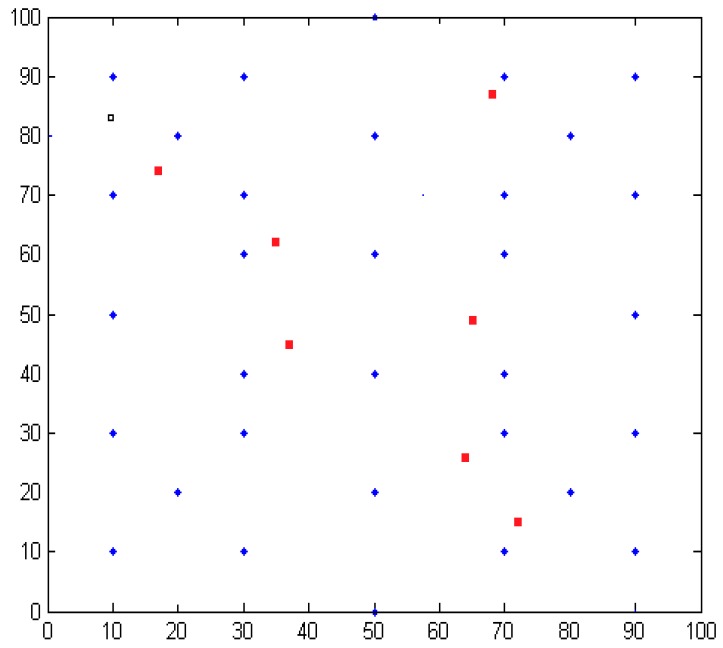
One of the simulation location area distribution maps.

Taking into account the different coverage of node communication, the following simulation environment is the region $100\times 100\;m^{2}$. The unknown node is evenly distributed in the positioning area. As shown in Figure 19, the red dots indicate the random distribution of the unknown nodes; blue points indicate the anchor nodes.

Taking the number of anchor nodes to be N=16,N=24,N=32 respectively, the location of the unknown node is computed. With the fluctuation of 5% or 10%, the unknown node is judged to be in a different location. According to the area of the unknown node, this may select the appropriate positioning algorithms, namely the unknown node inside or outside positioning unit method. Finally, part of the results for the positioning of unknown nodes are shown in Table 5, Table 6 and Table 7.

In the $100\times 100\;m^{2}$ positioning area, if a different number of anchor nodes are distributed, each anchor node area’s coverage is different, so the regional positioning division scope changes accordingly.

**Figure 20:**
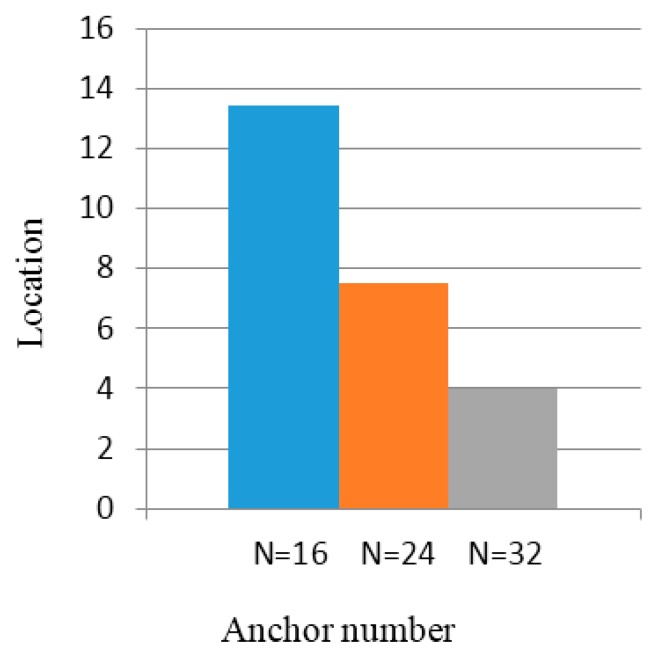
Location errors with intensity value fluctuation of 5%.

**Figure 21:**
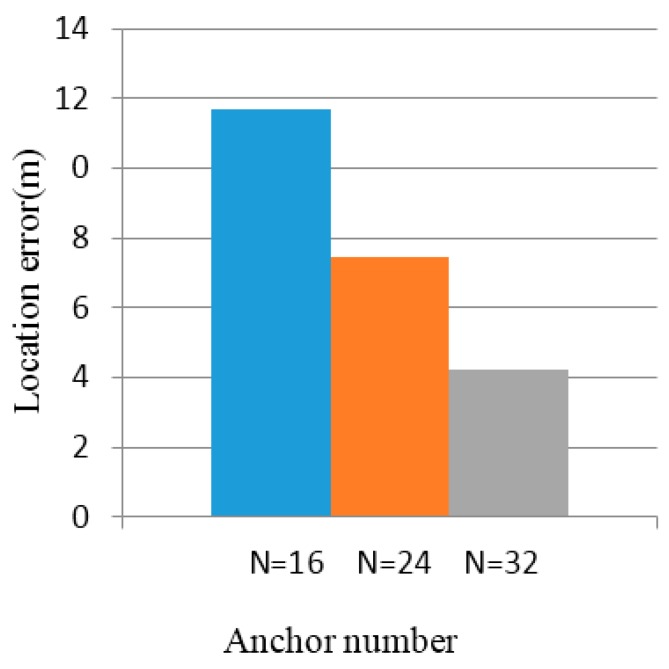
Location errors with intensity value fluctuation of 10%.

From Figure 20 and Figure 21, we see that the error of the unknown nodes decreases with the increase in the number of anchor nodes when the intensity data fluctuation is 5% and 10%. When the number of anchor nodes is 16, 24, or 32, the average location error of the unknown nodes is 12.55 m, 7.49 m, or 4.11 m. In the $100\times 100\;m^{2}$ regional location, because of the greater area of anchor nodes’ partitioning, the unknown node localization becomes larger, thus affecting the coordinates of unknown nodes. Compared to the effect of location, the positioning error is increased.

### 5.2. Experimental Results

The sensor node communication range is less than 25 m. In order to further study the feasibility, we use our localization algorithm in the real environment. In this paper, the ZigBee wireless sensor network system is used, which is provided by the Wireless Dragon Technology Company (Chengdu, China). The experimental equipment includes four anchor nodes, three unknown nodes, and gateway transmission equipment.

**Figure 22:**
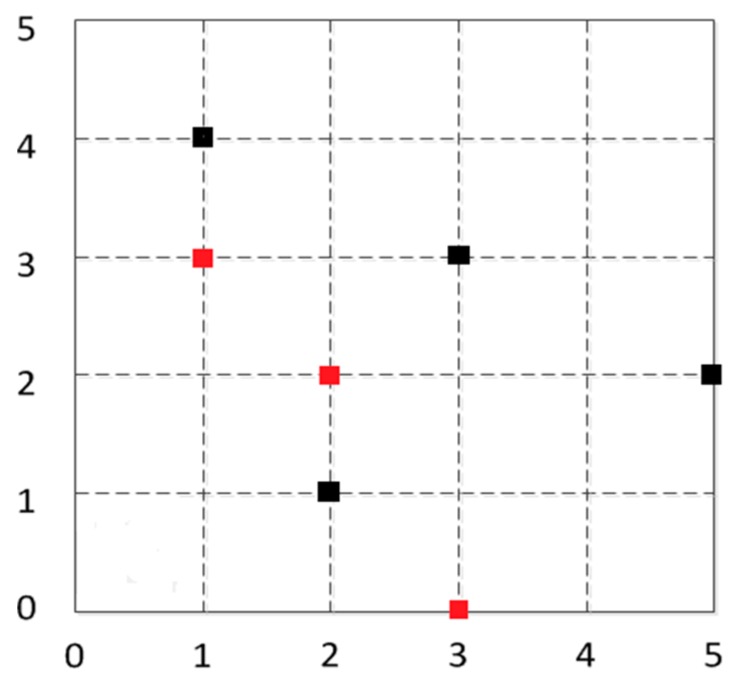
Experimental distribution map of location algorithm.

Figure 22 shows the experimental environment for the laboratory: in the $5\times 5\;m^{2}$ area of the four anchor nodes, the black squares are the anchor nodes, for which the coordinates are (2, 1), (5, 2), (3, 3), and (1, 4), respectively, and the red squares are the unknown nodes’ positions.

**Figure 23:**
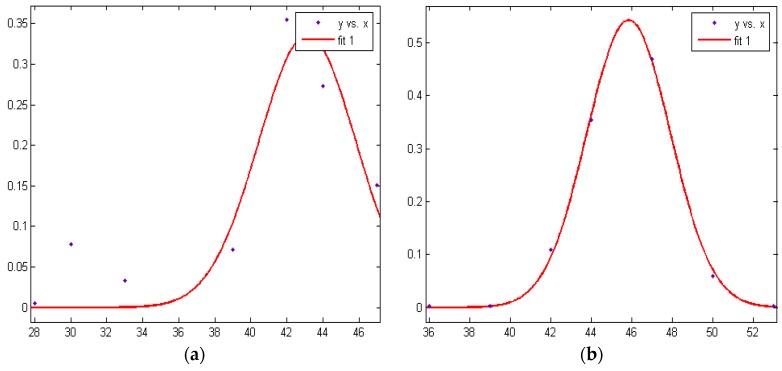
(**a**) Node 1 and (**b**) Node 4 3 meter RSSI fitting.

The main experimental process and steps are as follows:

Firstly, the anchor nodes are placed in a fixed position. The RSSI data are analyzed by Gaussian fitting. The peak value is selected as the most appropriate strength. Taking 3 meter intensity data between Node 1,Node 4 and the unknown nodes for example, shown in Table 8, these data calculate the probability density of each intensity, and the Gaussian fitting strength obtains the appropriate RSSI value, as shown in Figure 23.

Secondly, the wireless signal loss model is used to estimate the distance. Taking the original unknown node to be (1, 3) as an example, the strengths of the four anchor nodes are collected and the estimated distance values are as shown in Table 9.

**Figure 24:**
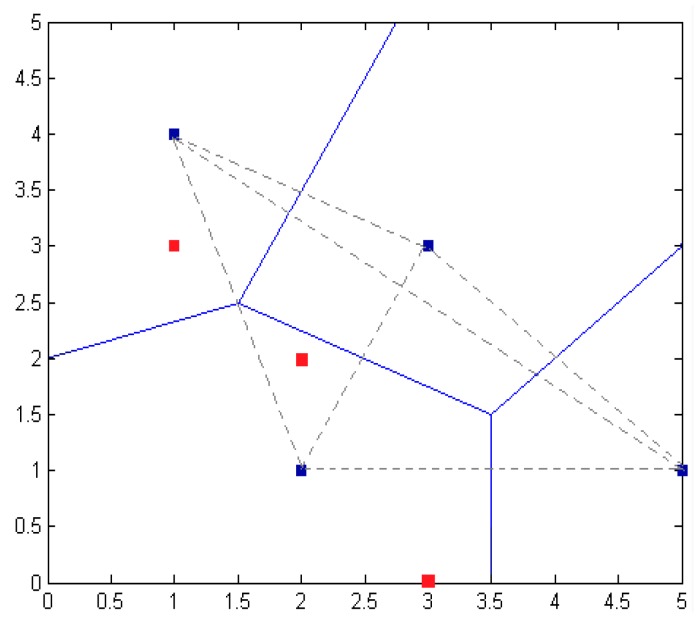
Sketch map of regional divisions.

Thirdly, for the regional division method, the whole positioning region is divided into polygons, and then the adjacent nodes are selected to be combined into a plurality of triangles. If the original coordinates of the unknown nodes are (1, 3), (2, 2), (3, 0), the nodes’ positioning unit areas are as shown in Figure 24: the blue solid line shows the anchor nodes dividing the central polygon region; the gray dotted area consists of neighboring nodes, shown as multiple triangles; the blue squares represent anchor nodes’ coordinates, and the red squares represent the original coordinates of the unknown nodes.

**Figure 25:**
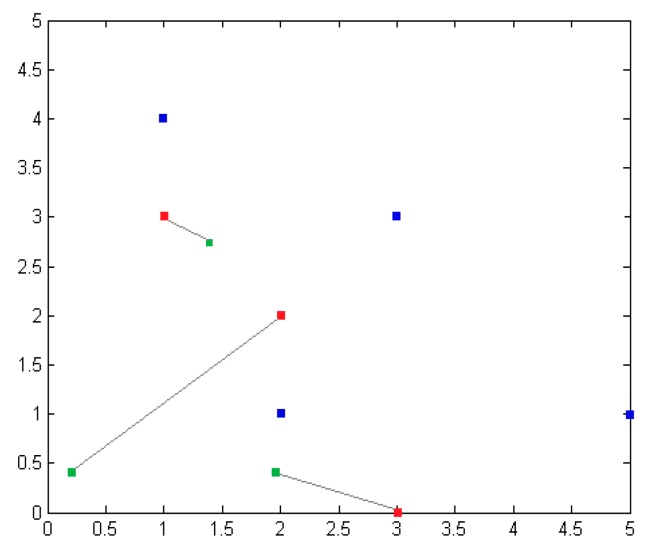
Node location map.

As shown in Figure 25, in the division of the location of the region, the blue squares represent the anchor nodes’ coordinates. The red squares represent the original coordinates of the unknown nodes. The green squares represent the unknown nodes’ coordinates. By measuring signal strength in the real environment, the model is fit to select a reasonable strength value. According to the positioning algorithm, the model selects the corresponding calculation method. Finally, we get unknown nodes in the $5\times 5\;m^{2}$ region, for which the average localization error is 1.089 m.

Because indoor interference factors cannot be absolutely avoided, the signal intensity error cannot be completely eliminated. Estimating the distance value will result in errors and the final node coordinate calculation data will be affected. The original coordinates of the unknown node are (1, 3), (2, 2), (3, 0), and (3, 2). Using this algorithm, the average coordinates and positioning error are calculated; the specific data are shown in Table 10.

## 6. Conclusions

In this paper, a ranging method based on RSSI—combined with multi-granularity regional division aiming at the area of the unknown nodes, Lagrange multiplication, and a Taylor expansion location algorithmis proposed and the algorithm is verified by simulations and experiments.

This paper researches the node localization algorithm based on RSSI. Intensity data collected are analyzed in the experiment. It is found that data change according to the different distances. The RSSI data values are basically in accord with normal distribution. Using a Gaussian fitting function, the intensity data collected are fitted, as far as possible, to eliminate interference and abnormal situations and choose a fitting peak that gives the optimal intensity values. Moreover, according to the multi-granularity localization, this paper establishes the dividing region method, that is, the overlapping area of the two polygons is regarded as the final positioning region, combining the Thiessen polygon and the triangle.

Different solutions are adopted to find the locations of the unknown nodes. When unknown nodes are inside positioning unit, a candidate virtual reference node is chosen using vector similarity. Three virtual reference points from the unknown node are selected and using the centroid algorithm we can calculate the final coordinates. When unknown nodes are outside of the positioning unit, the first step is to determine where the unknown node of the polygon area is. We also used the vector similarity method to select the appropriate virtual reference node. The Lagrange method was used to establish the calculation formula and substitute the preliminary coordinates into the Taylor series expansion. By gradually modifying the coordinates, reasonable coordinate values are finally acquired.

## Figures and Tables

**Table 1:** RSSI measurement range of four nodes at different distances.

Distance (m)	Node 1	Node 2	Node 3	Node 4
1	89~100	106~109	64~89	92~112
1.4	72~75	75~81	56~58	72~78
2	72~78	50~58	61~64	44~56
2.2	56~64	64~70	42~47	36~44
3	28~47	58~64	44~64	36~53
3.2	14~33	72~75	64	22~33
4	72	75~81	67~72	39~50
4.5	50~64	0~50	44~61	75~78

**Table 2:** Calculation results of *n* at different distances.

Node	1.4 m	2.2 m	3.2 m	4.5 m
Node 1	14.501	10.613	12.968	5.248
Node 2	19.545	11.544	6.731	11.973
Node 3	10.758	8.402	1.726	2.919
Node 4	18.053	17.262	15.003	3.735

**Table 3:** The localization results with RSSI fluctuating 5%.

Polygon Area Estimated	Actual V Area	Actual Triangle	Calculation Results of Coordinate
(7.0, 4.0)	(7.0, 6.0)	(7.0, 4.0) (7.0, 6.0) (5.0, 6.0)	(6.706, 5.226)
			(6.929, 5.254)
	(7.0, 4.0)		(6.833, 4.833)

**Table 4:** The localization results with RSSI fluctuating 10%.

Polygon Area Estimated	Actual V Area	Actual Triangle	Calculation Results of Coordinate
(7.0, 4.0)	(7.0, 4.0)	(7.0, 4.0) (7.0, 6.0) (5.0, 6.0)	(6.617, 4.967)
			(6.676, 5.314)
	(7.0 6.0)	(7.0, 4.0) (7.0, 6.0) (5.0, 4.0)	(6.061, 4.648)

**Table 5:** Localization results (*n* = 16).

Unknown Node Original Coordinates	The Calculated Coordinates of Intensity Fluctuation 5%	The Calculated Coordinates of Intensity Fluctuation 10%
(17, 74)	(21.903, 78.461)	(22.146, 73.352)
(37, 45)	(41.734, 25.467)	(41.888, 25.011)
(35, 62)	(26.790, 53.509)	(26.953, 52.599)
(64, 26)	(63.704, 23.186)	(63.796, 23.389)
(65, 49)	(58.850, 30.633)	(62.795, 43.769)
(68, 87)	(45.102, 76.509)	(45.103, 76.643)
(72, 15)	(67.238, 16.454)	(66.812, 16.079)
(17, 74)	(21.903, 78.461)	(22.146, 73.352)

**Table 6:** Localization results (*n* = 24).

Unknown Node Original Coordinates	The Calculated Coordinates of Intensity Fluctuation 5%	The Calculated Coordinates of Intensity Fluctuation 10%
(17, 74)	(21.58, 83.852)	(21.427, 83.947)
(37, 45)	(41.654, 25.246)	(41.847, 25.386)
(35, 62)	(39.206, 71.190)	(39.206, 71.190)
(64, 26)	(62.280, 23.889)	(65.416, 25.714)
(65, 49)	(62.899, 43.868)	(62.782, 43.935)
(68, 87)	(67.083, 85.833)	(68.333, 87.500)
(72, 15)	(66.746, 15.119)	(66.746, 15.119)
(17, 74)	(21.58, 83.852)	(21.427, 83.947)

**Table 7:** Localization results (*n* = 32).

Unknown Node Original Coordinates	The Calculated Coordinates of Intensity Fluctuation 5%	The Calculated Coordinates of Intensity Fluctuation 10%
(17, 74)	(14.699, 78.637)	(14.136, 78.586)
(37, 45)	(22.849, 42.435)	(36.666, 43.333)
(35, 62)	(33.333, 63.333)	(25.617, 59.021)
(64, 26)	(65.079, 25.714)	(61.220, 23.773)
(65, 49)	(63.333, 48.333)	(66.666, 48.333)
(68, 87)	(67.916, 86.666)	(67.222, 85.833)
(72, 15)	(72.500, 14.166)	(71.666, 13.333)
(17, 74)	(14.699, 78.637)	(14.136, 78.586)

**Table 8:** Collecting RSSI data of Node 1 and Node 4 at 3 m.

RSSI of Node 1 Measurement	RSSI Probability of Node 1	RSSI of Node 4 Measurement	RSSI Probability of Node 4
47	0.1511	53	0.0022
44	0.2733	50	0.0589
42	0.3544	47	0.4689
39	0.0711	44	0.3544
33	0.0333	42	0.1100
30	0.0778	39	0.0033
28	0.0056	36	0.0022

**Table 9:** Estimation results between node (1, 3) and anchor node.

Anchor Node Coordinates	Actual Distance (m)	Measuring Intensity	Estimating (m)
(5, 2)	4.12	60.41	2.505
(3, 3)	2.0	53.68	2.998
(1, 4)	1.0	72.72	1.801
(2, 1)	2.23	41.79	4.125

**Table 10:** Calculation results and error of unknown nodes.

Original Coordinates	Calculating Coordinate	Location Error	MLE Coordinate	Location Error
(1, 3)	(1.3844, 2.7378)	0.465	(0.5507, 2.9138)	0.4151
(2, 2)	(0.2140, 0.4107)	2.391	(3.2102, 1.5525)	1.291
(3, 0)	(1.9509, 0.4104)	1.127	(4.1510, 1.4900)	1.882
(3, 2)	(2.9304, 2.3687)	0.463	(5.0829, 2.5232)	2.876
